# Supplementary material for: Arioc: High-concurrency short-read alignment on multiple GPUs
Source: PLoS Comput Biol. 2020 Nov 9;16(11):e1008383. doi: 10.1371/journal.pcbi.1008383 (PMC7676696; doi:10.1371/journal.pcbi.1008383)
Supplement: S1 Text — (DOCX) [file pcbi.1008383.s001.docx]

Arioc: high-concurrency short-read alignment on multiple GPUs

Richard Wilton and Alexander S. Szalay

**S1 Text. GPU peer-to-peer memory interconnect topology**

The following tables represent GPU P2P memory-interconnect topology for the three computer configurations used in these experiments. The computers were provisioned with Nvidia V100 GPUs, each having 32GB of memory.

Direct peer-to-peer memory interconnect between each pair of GPUs is indicated in the tables as follows:

X Self

OK Supported

NS Not supported

For speed-versus-sensitivity experiments, only GPU devices 0, 1, 2, and 3 were used in each machine.

*Dell EMC HPC and AI Innovation Lab, Rattler cluster – gpuq partition, 4×V100 node*

|  | GPU 0 | GPU 1 | GPU 2 | GPU 3 |
| --- | --- | --- | --- | --- |
| GPU 0 | X | OK | OK | OK |
| GPU 1 | OK | X | OK | OK |
| GPU 2 | OK | OK | X | OK |
| GPU 3 | OK | OK | OK | X |

*PSC (Pittsburgh Supercomputing Center) Bridges – GPU-AI partition, DGX-2 node*

|  | GPU 0 | GPU 1 | GPU 2 | GPU 3 | GPU 4 | GPU 5 | GPU 6 | GPU 7 | GPU 8 | GPU 9 | GPU 10 | GPU 11 | GPU 12 | GPU 13 | GPU 14 | GPU 15 |
| --- | --- | --- | --- | --- | --- | --- | --- | --- | --- | --- | --- | --- | --- | --- | --- | --- |
| GPU 0 | X | OK | OK | OK | OK | OK | OK | OK | OK | OK | OK | OK | OK | OK | OK | OK |
| GPU 1 | OK | X | OK | OK | OK | OK | OK | OK | OK | OK | OK | OK | OK | OK | OK | OK |
| GPU 2 | OK | OK | X | OK | OK | OK | OK | OK | OK | OK | OK | OK | OK | OK | OK | OK |
| GPU 3 | OK | OK | OK | X | OK | OK | OK | OK | OK | OK | OK | OK | OK | OK | OK | OK |
| GPU 4 | OK | OK | OK | OK | X | OK | OK | OK | OK | OK | OK | OK | OK | OK | OK | OK |
| GPU 5 | OK | OK | OK | OK | OK | X | OK | OK | OK | OK | OK | OK | OK | OK | OK | OK |
| GPU 6 | OK | OK | OK | OK | OK | OK | X | OK | OK | OK | OK | OK | OK | OK | OK | OK |
| GPU 7 | OK | OK | OK | OK | OK | OK | OK | X | OK | OK | OK | OK | OK | OK | OK | OK |
| GPU 8 | OK | OK | OK | OK | OK | OK | OK | OK | X | OK | OK | OK | OK | OK | OK | OK |
| GPU 9 | OK | OK | OK | OK | OK | OK | OK | OK | OK | X | OK | OK | OK | OK | OK | OK |
| GPU 10 | OK | OK | OK | OK | OK | OK | OK | OK | OK | OK | X | OK | OK | OK | OK | OK |
| GPU 11 | OK | OK | OK | OK | OK | OK | OK | OK | OK | OK | OK | X | OK | OK | OK | OK |
| GPU 12 | OK | OK | OK | OK | OK | OK | OK | OK | OK | OK | OK | OK | X | OK | OK | OK |
| GPU 13 | OK | OK | OK | OK | OK | OK | OK | OK | OK | OK | OK | OK | OK | X | OK | OK |
| GPU 14 | OK | OK | OK | OK | OK | OK | OK | OK | OK | OK | OK | OK | OK | OK | X | OK |
| GPU 15 | OK | OK | OK | OK | OK | OK | OK | OK | OK | OK | OK | OK | OK | OK | OK | X |

*Amazon Web Services EC2 – p3dn.24xlarge instance*

|  | GPU 0 | GPU 1 | GPU 2 | GPU 3 | GPU 4 | GPU 5 | GPU 6 | GPU 7 |
| --- | --- | --- | --- | --- | --- | --- | --- | --- |
| GPU 0 | X | OK | OK | OK | OK | NS | NS | NS |
| GPU 1 | OK | X | OK | OK | NS | OK | NS | NS |
| GPU 2 | OK | OK | X | OK | NS | NS | OK | NS |
| GPU 3 | OK | OK | OK | X | NS | NS | NS | OK |
| GPU 4 | OK | NS | NS | NS | X | OK | OK | OK |
| GPU 5 | NS | OK | NS | NS | OK | X | OK | OK |
| GPU 6 | NS | NS | OK | NS | OK | OK | X | OK |
| GPU 7 | NS | NS | NS | OK | OK | OK | OK | X |
