# Supplementary material for: Arioc: High-concurrency short-read alignment on multiple GPUs
Source: PLoS Comput Biol. 2020 Nov 9;16(11):e1008383. doi: 10.1371/journal.pcbi.1008383 (PMC7676696; doi:10.1371/journal.pcbi.1008383)
Supplement: S2 Text — (DOCX) [file pcbi.1008383.s002.docx]

Arioc: high-concurrency short-read alignment on multiple GPUs

Richard Wilton and Alexander S. Szalay

**S2a Text. Arioc configuration parameters for WGS alignments**

<?xml version="1.0" encoding="utf-8"?>

<AriocP gpuMask="0x000F" batchSize="256K" verboseMask="0xE0000007">

<X useHinGmem="$(H)" useJinGmem="0" useHJinGPmem="$(HJ)" />

<R>/data/Arioc/R/GRCh38/enc</R>

<nongapped seed="ssi84_2_30" maxJ="200" maxMismatches="5" />

<gapped seed="hsi20_0_31" Wmxgs="2_6_5_3" Vt="L,0,1" maxJ="$(maxJ)" seedDepth="6" />

<Q filePath="/data/Arioc/Q/NCBI/SRP117159/SRR6020688/enc/">

<paired srcId="1" subId="1" srcInfo="NCBI:SRR6020688">

<file>SRR6020688_1</file>

<file>SRR6020688_2</file>

</paired>

</Q>

<A baseName="SRR6020688"

basePath="/nvme1/Arioc/A/NCBI/SRP117159"

overwrite="true"

pairFragmentLength="0-500"

pairOrientation="c"

pairCollision="oc">

<sam report="cdru">SRR6020688</sam>

</A>

</AriocP>

Parameter maxJ was varied to balance speed versus sensitivity. With higher maxJ settings, Arioc considers more locations in the reference genome as candidates for alignment and finds more higher-scoring mappings than it does when configured with lower maxJ settings. Throughput decreases and sensitivity increases with higher maxJ settings because more alignment computations are performed for each read:

|  | maxJ |
| --- | --- |
| greater throughput | 4 |
| . | 8 |
| . | 16 |
| . | 64 |
| . | 100 |
| greater sensitivity | 200 |

Parameters useHinGmem and useHJinGPmem were varied to specify lookup table memory layouts:

|  | useHinGmem | useHJinGPmem |
| --- | --- | --- |
| H and J in page-locked system RAM | 0 | 0 |
| H in GPU memory, J in page-locked system RAM | 1 | 0 |
| H and G in GPU memory | 0 | 1 |

**S2b Text. Arioc configuration parameters for WGBS alignments**

<?xml version="1.0" encoding="utf-8"?>

<AriocP gpuMask="0x000F" batchSize="256K" verboseMask="0xE0000007">

<X useHinGmem="$(H)" useJinGmem="0" useHJinGPmem="$(HJ)" />

<R>/data/Arioc/R/GRCh38/enc</R>

<nongapped seed="ssi84_2_30_CT" maxJ="200" maxMismatches="2" />

<gapped seed="hsi25_0_32_CT" Wmxgs="2_6_5_3" Vt="L,0,1" maxJ="$(maxJ)" seedDepth="3" />

<Q filePath="/data/Arioc/Q/NCBI/SRP117159/SRR6020687/enc/">

<paired srcId="1" subId="1" srcInfo="NCBI:SRR6020687">

<file>SRR6020687_1</file>

<file>SRR6020687_2</file>

</paired>

</Q>

<A baseName="SRR6020687"

basePath="/nvme1/Arioc/A/NCBI/SRP117159"

overwrite="true"

pairFragmentLength="0-500"

pairOrientation="c"

pairCollision="ocd">

<sam report="c">SRR6020687</sam>

<sam report="d">SRR6020687</sam>

<sam report="r">SRR6020687</sam>

<sam report="u">SRR6020687</sam>

</A>

</AriocP>

Parameter maxJ was varied to balance speed versus sensitivity:

|  | maxJ |
| --- | --- |
| greater throughput | 8 |
| . | 16 |
| . | 32 |
| . | 64 |
| . | 100 |
| greater sensitivity | 200 |

Parameters useHinGmem and useHJinGPmem were varied as for WGS alignments.
