# Supplementary material for: Arioc: High-concurrency short-read alignment on multiple GPUs
Source: PLoS Comput Biol. 2020 Nov 9;16(11):e1008383. doi: 10.1371/journal.pcbi.1008383 (PMC7676696; doi:10.1371/journal.pcbi.1008383)
Supplement: S3 Text — (DOCX) [file pcbi.1008383.s003.docx]

Arioc: high-concurrency short-read alignment on multiple GPUs

Richard Wilton and Alexander S. Szalay

**S3 Text. Bowtie 2 configuration parameters for WGS alignments**

-D $(D)

-R $(R)

-N 0

-L $(L)

-i $(i)

--ignore-quals

--local

--ma 2

--mp 6

--np 6

--rdg 5,3

--rfg 5,3

--score-min L,0,1

--minins 0

--maxins 500

--fr

--time

--xeq

--threads 48

Parameters D, R, L, and i were varied to balance speed versus sensitivity by setting the number of reference-sequence locations at which alignments were computed for each read:

|  | D | R | L | i |
| --- | --- | --- | --- | --- |
| very fast local | 5 | 1 | 25 | S,1,2.00 |
| sensitive local | 15 | 2 | 20 | S,1,0.75 |
| very sensitive local | 20 | 3 | 20 | S,1,0.50 |
| (greater sensitivity) | 100 | 3 | 20 | C,1,0 |
