# Supplementary material for: Arioc: High-concurrency short-read alignment on multiple GPUs
Source: PLoS Comput Biol. 2020 Nov 9;16(11):e1008383. doi: 10.1371/journal.pcbi.1008383 (PMC7676696; doi:10.1371/journal.pcbi.1008383)
Supplement: S4 Text — (DOCX) [file pcbi.1008383.s004.docx]

Arioc: high-concurrency short-read alignment on multiple GPUs

Richard Wilton and Alexander S. Szalay

**S4 Text. Bismark configuration parameters for WGBS alignments**

--parallel 10

-p 2

-L 25

--minins 0

--maxins 500

--score_min G,150,0

--local

Settings for –parallel and –p were chosen empirically using a one million pair subset of the reads in WGBS sample SRR6020687. The setting for --score_min specifies the same minimum alignment score threshold for this data as the L,0,1 parameter value used with Arioc and Bowtie 2.
