# Supplementary material for: Arioc: High-concurrency short-read alignment on multiple GPUs
Source: PLoS Comput Biol. 2020 Nov 9;16(11):e1008383. doi: 10.1371/journal.pcbi.1008383 (PMC7676696; doi:10.1371/journal.pcbi.1008383)
Supplement: S5 Text — (DOCX) [file pcbi.1008383.s005.docx]

Arioc: high-concurrency short-read alignment on multiple GPUs

Richard Wilton and Alexander S. Szalay

**S5a Text. Correct versus incorrect mappings, classified by MAPQ (error rate 0.25%)**

Correct versus incorrect mappings, classified by MAPQ, for Arioc, Bowtie 2, and SOAP3-dp, using 10,000,000 150nt paired-end reads (20,000,000 mates) simulated from GRCh38.p12 with mason2 v2.0.9 (https://github.com/seqan/seqan).

Non-default settings for mason2:

--illumina-read-length 150

--illumina-prob-insert 0.00005

--illumina-prob-deletion 0.00005

--illumina-prob-mismatch 0.0024

All aligners configured with scoring parameters as follows:

Match: +2

Mismatch: -6

Gap open: -5

Gap space: -3

Minimum alignment score: 225

Local alignments, base quality scores ignored

Favor sensitivity over speed (see S2a Text and S3 Text)

**S5b Text. Correct versus incorrect mappings, classified by MAPQ (error rate 7.0%)**

Correct versus incorrect mappings, classified by MAPQ, for Arioc, Bowtie 2, and SOAP3-dp, using 10,000,000 150nt paired-end reads (20,000,000 mates) simulated from GRCh38.p12 using mason2 v2.0.9 (https://github.com/seqan/seqan).

Non-default settings for mason2:

--illumina-read-length 150

--illumina-prob-insert 0.005

--illumina-prob-deletion 0.005

--illumina-prob-mismatch 0.06

All aligners configured with scoring parameters as follows:

Match: +2

Mismatch: -6

Gap open: -5

Gap space: -3

Minimum alignment score: 150

Local alignments, base quality scores ignored

Favor sensitivity over speed (see S2a Text and S3 Text)
