# Supplementary material for: Arioc: High-concurrency short-read alignment on multiple GPUs
Source: PLoS Comput Biol. 2020 Nov 9;16(11):e1008383. doi: 10.1371/journal.pcbi.1008383 (PMC7676696; doi:10.1371/journal.pcbi.1008383)
Supplement: S6 Text — (DOCX) [file pcbi.1008383.s006.docx]

Arioc: high-concurrency short-read alignment on multiple GPUs

Richard Wilton and Alexander S. Szalay

**S6 Text. Arioc hardware resource utilization**

Arioc allocates hardware resources dynamically. Resource consumption depends on a variety of factors, including

- the size and layout of reference-genome lookup tables
- the number of GPU devices in use
- the average length of read sequences
- the configured number of reads in each batch
- the number of Smith-Waterman alignments computed for each read batch

Arioc uses CPU and GPU resources concurrently:

- CPU threads to manage file input and output, to sort and prioritize mappings for output, and to compute alignment metadata such as MAPQ
- CPU-addressable system memory buffers to initialize and contain lookup tables, load read sequence data and transfer it between CPU and GPU memory buffers, format alignment results, and buffer formatted results for output
- GPU memory buffers to contain lookup tables, sort and prioritize candidate reference-sequence locations, and compute alignments

Arioc always uses all available GPU memory, but its utilization patterns for other resources vary according to read sequence data and runtime configuration parameters. Arioc uses CPU memory in proportion to the size of its lookup tables, so its overall system RAM utilization is greater than that of CPU-only aligners such as Bowtie 2 that use compressed index structures.

Dynamic utilization can be monitored with tools such as Windows Resource Monitor, Linux top, and nvidia-smi. With these tools, here are some representative resource-utilization values for Arioc, SOAP3‑dp, and Bowtie 2:

|  |  | CPU threads^1^ | GPU threads^2^ | system RAM | | GPU RAM | |
| --- | --- | --- | --- | --- | --- | --- | --- |
|  |  |  |  | initializing | aligning | total | LUTs |
| Arioc | H, J in system RAM | 1500% | 95% | 77.8GB | 77.8GB | 31.7GB | 2.2GB |
|  | H in GPU memory | 1500% | 95% | 80.1GB | 57.3GB | 31.7GB | 27.2GB |
|  | H, J in GPU memory | 1500% | 95% | 79.9GB | 4.6GB | 31.7GB | 21.7GB |
| SOAP3-dp |  | 190% | 95% |  | 22.5GB | 1.4GB |  |
| Bowtie 2 |  | 3990% |  |  | 6.5GB |  |  |

^1^ 100% = one fully utilized CPU thread
^2^ 100% = all available GPU threads

Data: 10,000,000 simulated 150nt paired-end WGS reads from GRCh38.p12 (same as S5b Text)
Hardware: Dell C4140 (40 CPU threads, 384GB RAM, 4 Nvidia V100 32GB GPUs)
