# Supplementary material for: Arioc: High-concurrency short-read alignment on multiple GPUs
Source: PLoS Comput Biol. 2020 Nov 9;16(11):e1008383. doi: 10.1371/journal.pcbi.1008383 (PMC7676696; doi:10.1371/journal.pcbi.1008383)
Supplement: S1 Table — (DOCX) [file pcbi.1008383.s007.docx]

Arioc: high-concurrency short-read alignment on multiple GPUs

Richard Wilton and Alexander S. Szalay

**Table T1. Representative Nvidia GPU devices since 2012**

This list contains one representative of each Nvidia GPU microarchitecture offered since 2012.

Peer-to-peer memory interconnect between multiple GPUs using a dedicated hardware path (NVlink) was not supported prior to the Pascal microarchitecture.

| model | year | microarchitecture | on-device RAM | threads | peer access |
| --- | --- | --- | --- | --- | --- |
| K20 | 2012 | Kepler | 5GB | 26624 | no |
| M40 | 2015 | Maxwell | 16GB-24GB | 49152 | no |
| P100 | 2016 | Pascal | 16GB | 114688 | NVlink 1.0 |
| V100 | 2018 | Volta | 16GB-32GB | 163840 | NVlink 2.0+ |
