# Supplementary material for: Arioc: High-concurrency short-read alignment on multiple GPUs
Source: PLoS Comput Biol. 2020 Nov 9;16(11):e1008383. doi: 10.1371/journal.pcbi.1008383 (PMC7676696; doi:10.1371/journal.pcbi.1008383)
Supplement: S2 Table — (DOCX) [file pcbi.1008383.s008.docx]

Arioc: high-concurrency short-read alignment on multiple GPUs

Richard Wilton and Alexander S. Szalay

**Table T2. Short-read aligner versions and distribution websites**

| software | version | distribution |
| --- | --- | --- |
| Arioc | 1.40 | https://github.com/RWilton/Arioc |
| Bowtie 2 | 2.3.5.1 | https://sourceforge.net/projects/bowtie-bio/files/bowtie2/2.3.5.1 |
| Bismark | 0.22.3 | https://github.com/FelixKrueger/Bismark |
