# Supplementary material for: Arioc: High-concurrency short-read alignment on multiple GPUs
Source: PLoS Comput Biol. 2020 Nov 9;16(11):e1008383. doi: 10.1371/journal.pcbi.1008383 (PMC7676696; doi:10.1371/journal.pcbi.1008383)
Supplement: Attachment — Submitted filename: Response to Reviewers.3.pdf [file pcbi.1008383.s010.pdf]

Response to Reviewers

Manuscript ID: PCOMPBIOL-D-20-00150
Title: Arioc: high-concurrency short-read alignment on multiple GPUs
Date: 5 June 2020

To each of our reviewers --

We thank you both for taking the time to evaluate and criticize our manuscript. We have carefully considered your comments. We have incorporated most of your suggestions into our manuscript and, in a few cases, explained why we elected not to change things.

Our responses to each reviewer follow on separate pages of this document.

Richard Wilton

Response to Reviewer 1

General comments
Authors present a new implementation of their short-read aligner Arioc.

The proposed implementation takes advantage of the most recent GPU hw architecture supporting high-bandwidth P2P memory access among multiple GPUs to increase its performance in terms of computing time.

Although the work is interesting there are several points that need to be clarified.

Major comments
1. My main concern is related to the results.

1.a - Authors did not perform experiments on simulated libraries with the aim to assess the reliability of the new version of ARIOC. These analyses were carried out with previous versions of the tool. However, as this is a new implementation it must be validated. Authors should perform these experiments and compare the results with those of the other tools. Simulated libraries should also be permanently accessible through a DOI or according to the journal policies.

> Response: We have added these validation results on page 4 of the manuscript ("Software design and implementation"), with a callout to results in the Supplemental Data (Appendix A5a and A5b).

1.b - Experiments on real data are not complete. For instance, for Bowtie2 results were only reported for the library SRR6020688. Experiments must be performed for all libraries.

> Response: The results actually do represent experiments for all the possible combinations of software and sequencing samples (libraries). We evaluated Arioc with both whole-genome sequencing (WGS) and whole-genome bisulfite sequencing (WGBS) samples, Bismark with WGBS samples, and Bowtie 2 with WGS samples. Bismark does not align WGS data, nor does Bowtie 2 align WGBS data.
>
> We took your comment to heart, however, and realized that we had not described the capabilities of each read aligner in regard to WGS and WGBS data. We addressed this by adding specifics to the paragraph headed "Comparisons with CPU-only software" (page 6) and by replacing sample accession IDs with descriptive headings in the chart graphics in the Supplemental Data.

1.c - Unbalanced hardware configurations were used to analyze the performance ARIOC and the other tools. For instance using AWS (Suppl Data D2) ARIOC was run using 4 NVIDIA V100 and 96 threads whereas Bowtie 2 was run using 40 threads. Experiments should be performed using an identical hardware configuration and using modern processors supporting hundreds of cores. A comparison between Bowtie2/Bismark and ARIOC with a single GPU and identical modern processor should also be performed.

> Response: This comment highlights two interesting issues. One is the difficulty of comparing the performance of CPU-only and GPU-accelerated software. The other involves comparing the dollar cost of using software

that accomplishes the same processing task using very different hardware resources in a commercial cloud.

The former problem has been investigated in previous publications, including [Wilton et al. 2015] and [Langmead et al. 2019]. Specifically, Arioc's throughput increases with additional GPU threads, but since the software uses CPU threads only for managing file input/output and computing metadata such as MAPQ scores, additional CPU threads have no significant effect on overall throughput. This can incidentally be seen in the current results, where speeds in Dell's HPC lab (40 CPU threads) are similar to those obtained on an Nvidia DGX-2 (96 CPU threads).

In contrast, Bowtie's throughput scales almost ideally with the number of available CPU threads on commonly used CPUs with up to 64 threads, although thread contention for hardware resources limits throughput with CPU thread counts in the "experimental" range of 96 or more. In the now-extinct Xeon Phi architecture, with up to 272 concurrent threads, throughput was still an order of magnitude lower than we see with Arioc on four GPUs [Langmead et al. 2019].

As for performance comparisons in a commercial cloud: given the performance uncertainties associated with hardware virtualization, cloud load balancing, and so on, we focused on comparing the dollar cost of using CPU-only and GPU-accelerated software. Our comparison was simplified by the fact that AWS per-hour prices for CPU resources are directly proportional to the number of CPU threads in use; since Bowtie's throughput (and hence the total execution time) scales with the number of CPU threads available, the dollar cost varies little with regard to the number of CPU threads in an AWS instance.

We did not repeat earlier performance comparisons on single-GPU configurations because single-GPU performance cannot be directly compared to multiple-GPU performance with high-bandwidth GPU memory interconnect. Both Arioc and Bowtie 2 have evolved in the five years since we performed a detailed performance comparison, and informally we still observe an order-of-magnitude speed difference between Arioc on an Nvidia V100 and Bowtie 2 on a 64-thread CPU. But repeating the in-depth head-to-head performance comparison we published in 2015 is beyond the scope of the current work.

1.d - As done for Bowtie2 and Bismark comparative experiments should also be performed for SOAP3-dp with the aim to confirm the best performances of ARIOC. As a reader, I would be curios to read about the behaviour of SOAP3-dp with the new V100 equipped with 32GB of memory. Also in this case experiments should be performed using an identical hardware configuration.

Response: We, too, would have liked to carry out a head-to-head comparison with SOAP3-dp, a GPU-accelerated aligner whose single-GPU performance approaches that of Arioc in some respects. Unfortunately, we were unable to obtain speed-versus-sensitivity metrics for SOAP3-dp because there are no user-configurable parameters that vary the amount of computation that the aligner performs in searching for high-scoring alignments.

Although SOAP3-dp is not designed to use multiple GPUs, it might have been possible to execute the program concurrently on multiple GPUs by splitting FASTQ input into multiple partitions, executing separate independent

instances of the aligner, combining the resulting output files, and computing aggregate statistics (overall mapping efficiency, mean TLEN, and so on). But the amount of programming required to "wrap" SOAP3-dp in this way would be significant.

Nevertheless, we were able to carry out a comparison between Arioc and SOAP3-dp in terms of accuracy as described above.

1.e - Results in supplementary data (D1 - D5) should be report the same (common) information for all tools. For instance, the table reporting results for Bowtie2 (Suppl Data D2) reports a column labeled "overall mapped %", but the same column is not present in tables for Arioc. Moreover, for an easy reading of the results, each column of the tables should be described.

Response: We updated worksheets D1, D2, D3, and D4 to report comparable numbers for Arioc, Bowtie, and Bismark with the same column headings and column positions in each worksheet. We also removed the extraneous "overall mapped %" column we had included in worksheet D2.

We also updated the Table of Contents worksheet with descriptions of each of the comparable column headings in these worksheets.

1.f - In Supplementary Data D1 are reported performance obtained for an unpublished library "LIBD1373". I don't think the library is mentioned in the article nor is it downloadable from the ARIOC repository at https://github.com/rwilton/arioc.

Response: These results were obtained in the context of as-yet-unpublished research. We had anticipated that this data would have been placed in the public domain prior to publication of the current manuscript, but since this has not yet occurred we are unfortunately unable to include it among our current results. It has been removed from the Supplementary Data.

1.g - Figure 4 show speed vs sensitivity for the LUT layouts. I observe that increasing the sensitivity (par. maxJ) the speed tends to converge for all LUT layouts. It would seem that by increasing the sensitivity there are not more advantages related to the device memory and NVLINK.

Response: All short-read aligners exhibit an exponential drop-off in speed as sensitivity is maximized. We assume that this reflects the amount of work an aligner must perform in searching for mappings for read sequences that contain multiple differences from the reference genome. Indirect evidence of this may be seen in the "candidates per read" metric which estimates the average number of Smith-Waterman dynamic programming problems computed per read and which increases by 8x as we maximize sensitivity in our experiments.

You are correct that the speed increase due to GPU memory interconnect is eventually outweighed by the computational burden of searching for additional mappings for hard-to-align read sequences at the upper limits of sensitivity. In practice, of course, short-read aligners are rarely (if ever) used at this extreme limit of sensitivity because the small additional percentage of properly aligned reads consists of these hard-to-align reads with lower alignment scores and mapping quality. When we evaluate performance, however, we always determine where the high-

sensitivity drop-off occurs so that optimal speed-versus-sensitivity settings become apparent.

(Incidentally, we find that visualizing the relationship between speed and sensitivity is essential to evaluating the performance of any short-read aligner. Almost any short-read aligner can appear "faster" than any other if one compares speeds selected from different parts of the speed-versus-sensitivity curve. We avoid this trap by measuring and visualizing each aligner's characteristic speed-versus-sensitivity behavior.)

1.h - Can you also comment about the overall host (and device) memory consumption? Is the host (device) memory consumption comparable with Bowtie2/Bismark/SOAP3-dp?

Response: This is not an easy question to answer. Arioc acquires CPU and GPU hardware resources dynamically according to its runtime parameterization and the number and capabilities of GPU devices. It then does as much parallel computation as these constraints allow.

We have added a new Appendix A6 to the Supplementary Information that provides more detail as well as a comparison with SOAP3-dp and Bowtie 2.

2. All experiments were carried out with the human genome. Today the scientific community is also heavily involved in the study of more complex genomes. My question is whether ARIOC and its LUT are suitable for very big and highly repetitive genomes.

Response: Arioc supports reference genomes up to 2^{34} base pairs (17G base pairs) in size. The largest reference genome we have used with it is the current release of T. aestivum (bread wheat), a highly repetitive 15gbp genome – but unfortunately its Arioc lookup tables are proportionately larger and too big to fit into GPU memory with a 4-GPU system, so we could not include it in our current results. With the T. aestivum genome, Arioc is still fast (300,000 reads/second on the same Dell HPC computer we used to measure performance with GRCh38) but we will have to wait until Nvidia releases a GPU with 64GB of device memory before we can generate comparable 4-GPU results for T. aestivum.

3. Line 41: "The first step in analyzing short-read DNA sequencer data is read alignment …" It would be more correct to write that alignment is at the base of the NGS analyses. Quality control and filtering are mandatory steps before alignment.

Response: Good point. We have reworded this assertion.

4. Finally, I suggest (it is not mandatory) to dockerize the tool installation and deposit it on DockerHub and/or BioContainers. Containers allow a fast deployment on local clusters as well as on the cloud.

Response: This is a good idea. We have hesitated to do it because of the wide variety of GPU hardware configurations and system environments to which a containerized installation would need to adapt.

In this context, Arioc is more of a Ferrari than a Volkswagen: we assume that its users will spend a few hours configuring, tuning, and optimizing its performance before using it on hundreds or thousands of WGS samples.

We are concerned that a containerized distribution would complicate rather than simplify this process.

For example, one performance-tuning strategy is to build from source code with a specific GPU hardware target in order to optimize the compiled executable binary. Another is to encode the reference-genome lookup tables with 32-bit hash values instead of 30- or 31-bit hash values. These techniques depend on GPU and host-system hardware configurations that cannot easily be generalized in a containerized distribution.

Minor comments

Labels “Figure 2” and “Figure 3” refer to tables.

Response: We have renamed these "figures" as "tables" and renumbered the remaining figures accordingly.

Line 58: (For example, these LUTs can occupy up to …): remove the brackets

Response: Done.

Response to Reviewer 2

General comments

This manuscript addresses an important area of short-read alignment. A new version of Arioc has been developed by utilising the multiple GPUs on the same machine to achieve a substantial speed up in short-read alignment. This new version of Arioc can benefit from larger GPU memories and high-bandwidth peer-to-peer memory access between GPUs. The manuscript is well written. I have a few comments:

The authors should explicitly mention that this is a newer version of Arioc.

> _Response_: We have updated the section labeled "Software design and implementation" (page 4) to point this out.

According to the results, Arioc has a better performance compared with the other aligners like Bowtie2 (for WGS) and Bismark (for WGBS) in terms to accuracy and running time. It is understandable that Arior performs faster due to the utilisation of multiple GPU technology. However, why does Arior have a better accuracy? Is it due to the algorithm used in Arioc? The authors should briefly explain this in the manuscript.

> _Response_: We agree with your observations about Arioc's performance! But when we use MAPQ (mapping quality) to evaluate accuracy (Supplemental Appendix A5a and A5b), there is little difference among the read aligners we evaluated. All three aligners are highly sensitive and specific, especially at the low error rates typical of current Illumina sequencers.
>
> Compared to Bowtie 2 and Bismark, Arioc does tend to find proper mappings for a small additional percentage of reads in a given WGS or WGBS sample. This is not due to algorithmic differences per se, but because Arioc computes alignments at more reference-sequence positions per read than the comparable CPU-only aligners. As we described in our original publication, we built Arioc using algorithms and implementations that are well suited to execution on GPU hardware, both to efficiently prioritize candidates for alignment and to compute many alignments in parallel.
>
> This relative increase in sensitivity is generally attributable to a small subset of reads that have multiple differences from the reference sequence and that are consequently reported with low alignment scores and low mapping quality scores. Such mappings may be useful (for example, in searching for potential structural variants) but often they are simply discarded during subsequent processing due to their low AS and MAPQ.

The authors need to explain what "concordant" alignment means. This is important in order to understand how the accuracies of different aligners were evaluated.

> _Response_: We have added a description of the criteria for proper (concordant) mappings. See page 5 near line 110.

Figure 5 mentions "concordantly-mapped pairs". Does this figure only consider the aligned pairs? How about the case that one of reads in the pair is properly aligned but another cannot? If Figure 5 only considers the aligned pairs, it is necessary to have another figure showing, for different aligners, the %

of reads concordantly aligned, including those either with only one read or both reads in the pairs being aligned.

> *Response*: [Figure 5 in the previous revision of this manuscript is now Figure 3.]
>
> Your observations again point out that the manuscript was unclear about the concept of "proper" or "concordant" mappings. We have addressed that by clearly stating the meaning of that terminology as noted above.

This manuscript does not mention about the alignment algorithm used in Arioc. The authors should briefly mention this and refer to their previous papers for details.

> *Response*: We have added a brief description of the alignment algorithms and cited the original published discussion. (See page 4, "Software design and implementation").
