# Supplementary material for: Arioc: High-concurrency short-read alignment on multiple GPUs
Source: PLoS Comput Biol. 2020 Nov 9;16(11):e1008383. doi: 10.1371/journal.pcbi.1008383 (PMC7676696; doi:10.1371/journal.pcbi.1008383)
Supplement: Attachment — Submitted filename: Response to Reviewers.4.docx [file pcbi.1008383.s011.docx]

**Response to Reviewers**

**Manuscript ID:**	PCOMPBIOL-D-20-00150

**Title:**	Arioc: high-concurrency short-read alignment on multiple GPUs

**Date:**	15 August 2020

To each of our reviewers --

We thank you both again for evaluating the manuscript changes we made in response to your initial review of our manuscript.

Our responses follow on separate pages of this document.

Richard Wilton

**Response to Reviewer 1**

*General comments*

Although the authors have worked to improve the manuscript, I must point out that some of my indications have not been fully implemented.

In the following I report my comments to the authors' responses.

1.a. The authors performed tests on synthetic data as required. However, they did not provide a link to download the simulated libraries as required. Readers must be able to reproduce the experiments. Please, provide a DOI to share the libraries with the readers.

*Response*: We have uploaded copies of this data to a public repository, figshare.com. (See "Future directions and availability", near line 247.) The tentative DOI is 10.6084/m9.figshare.12781298. The data will become public when it can be linked to the published article.

1.c. Considerations made by the authors are interesting and should be reported in the manuscript. However a comparison of the performance in terms of computing time must be based on an identical hw configuration. I have no doubt that ARIOC outperforms the other tool, but what is the information given by performances obtained with unbalanced hw configuration for different tools?

*Response*: As reviewers of other people's software as well as developers of our own software tools, we appreciate the notion of using a common hardware baseline to measure the performance of comparable software implementations. We have therefore added speed-vs-sensitivity values for Bowtie 2 using the same computer we used for evaluating Arioc, with the same WGS data (SRR6020688), at the Dell HPC lab (Supplemental Data D1).

Nevertheless, the gist of our current work is not to repeat earlier head-to-head comparisons between Arioc and other short read aligners but to demonstrate that modern GPU memory architecture can be exploited to obtain a significant improvement in speed. For this reason, the salient performance evaluations are those that compare the configurable GPU memory layouts within Arioc (Figures 1 and 2).

To respond more directly to the question regarding "unbalanced" hardware configurations: When new software implements only a new algorithmic approach or more efficient engineering techniques, it makes sense to evaluate its performance on the same hardware platform as existing software. But cross-platform GPU-to-CPU performance evaluations are highly problematic^[1]^.

GPU-versus-CPU comparisons depend not only on GPU device capabilities and on CPU thread counts and clock speeds but also on nondeterministic factors such as dynamic throttling of clock speeds with increased ambient temperature, peak I/O bandwidth (as suggested in Supplemental Data D5), and so on. Although using a single machine for performance evaluations might mitigate the effect of some of these variables, it is not possible to define speed or memory-usage "equivalence" or "balance" between software executing on GPU hardware and software running only on CPU threads.

Our results clearly support the assertion that Arioc's GPU-accelerated implementation executes an order of magnitude faster than CPU-only implementations on several different configurations of GPU devices and CPUs. Furthermore, by observing the dollar cost of using commercial cloud computer configurations that are optimal for each aligner, we can provide comparisons that have tangible implications for potential users. This is practical information for anyone choosing among these software tools for large-scale WGS or WGBS processing.

1.g. Please report in the manuscript the considerations given in your reply to the author.

*Response*: We have added this to the discussion on page 7 near line 184.

1.h. As requested, the authors report a comparison in terms of memory consumption between ARIOC and the other tools. The results of the comparison are reported in Appendix A6. However, no discussion of memory consumption is provided in the manuscript as well as no reference is made to Appendix A6. Please, discuss in the manuscript about the performances in terms of memory consumption. What considerations about the high RAM required by ARIOC with respect to the other tools?

*Response*: We have included a comment on comparative memory utilization as well as a callout to Appendix A6 (see page 6 near line 160). We also added a brief comment to Appendix A6 itself.

2. Please report in the manuscript the considerations given in your reply to the author. Constraints as the maximum genome size as well as other limitations to use ARIOC must be reported and discussed in the manuscript. This information is very important for researchers that plan to use a tool.

*Response*: We have revised the discussion of our results, in the section entitled "Advantages and limitations" (page 6 near line 172), to mention the maximum reference genome size supported by Arioc. We have also noted that the amount of available GPU memory can limit the use of a partitioned LUT memory layout to obtain maximum speed.

4. As wrote in my previous comment, I only suggested to dockerize ARIOC. I can understand the difficulties of building an optimized container. In any case, using your Ferrari/Volkswagen comparison, observe that only few people drive a Ferrari whereas a lot drive a Volkswagen. Since the installation and configuration take hours, the risk is that an unskilled user will abandon the tool in favour of others that are easy to install. For the future, I suggest to take into account the possibility to build a container for ARIOC.

*Response*: We agree.

Our plan is to obtain some practical measurements of Arioc's throughput with several hundred WGS samples in the new Expanse cluster at SDSC, where fifty 4xV100 compute nodes will be available, each configured much like the machine we used for the current article at Dell's HPC laboratory.

This will present an excellent opportunity for us to work out the details of containerizing the Arioc executables and associated index structures. We hope also to compare this with the current approach in which the Arioc binaries are made available through lmod and the associated lookup table files are placed in a shared public filesystem.

**Response to Reviewer 2**

(no additional comments)

1. See for example: https://www.usenix.org/legacy/events/hotpar11/tech/final_files/Anderson.pdf
